# Toxicological Evaluation of a Polyherbal Formulation (18KHT01) and Validation of UPLC-DAD Method for Quality Control

**DOI:** 10.1155/2024/1767618

**Published:** 2024-09-19

**Authors:** Prakash Raj Pandeya, Ramakanta Lamichhane, Gopal Lamichhane, Kyung-Hee Lee, Hyun-Ju Jung

**Affiliations:** ^1^ Department of Oriental Pharmacy and Wonkwang-Oriental Medicines Research Institute Wonkwang University, Sinyong-Dong, Iksan 570-749, Republic of Korea; ^2^ Department of Animal and Food Sciences University of Kentucky, Lexington, Kentucky 40546, USA; ^3^ Department of Pharmacy Kathmandu University, Dhulikhel 45200, Nepal; ^4^ Department of Nutritional Sciences Oklahoma State University, Stillwater, Oklahoma 74078, USA

**Keywords:** 18KHT01, acute toxicity, method validation, polyherbal formulation, subacute toxicity, UPLC-DAD

## Abstract

**Background:** 18KHT01 is a novel synergistic composition of *Quercus acutissima*, *Camellia sinensis*, *Geranium thunbergii*, and a small portion of *Citrus limon*. Our previous report demonstrated that the 18KHT01 exhibits potent antiobesity effects, with synergistic antioxidant, antiadipogenic, and antiobesity activities in diet-induced obese mice. This study explores the toxicity profile and quality control parameters of the 18KHT01 formulation.

**Methods:** Broad-spectrum acute and subacute oral toxicity studies were performed using male and female ICR mice. In order to simultaneous analysis of the 18KHT01 formulation, an ultraperformance liquid chromatography coupled with a diode array detector (UPLC-DAD) method was developed and validated using six marker compounds.

**Results:** Acute oral toxicity evaluation of 18KHT01, administered at single high doses of 2, 2.5, 3, and 5 g/kg, identified 2 g/kg as the no-observed adverse effect level (NOAEL). The LD50 (50% lethal dose) and LD100 (100% lethal dose) of 18KHT01 for male ICR mice were 3.99 and 7.77 g/kg, and those for the female mice were 2.94 and 4.70 g/kg, respectively. In addition, a 30-day repeated dose oral subacute toxicity evaluation indicated that 18KHT01 is safe below 500 mg/kg/day for long-term administration in ICR mice of either sex. UPLC-DAD method validation revealed that each calibration curve for the marker compounds showed good linearity, as well as the validation parameters such as precision, specificity, and accuracy met the acceptance criteria.

**Conclusion:** The present study evidenced the toxicological profile of 18KHT01 polyherbal formulation in mice as well as developed a simple, rapid, and accurate chromatographic method for quality control.

## 1. Introduction

A polyherbal formulation is the combination of several medicinal herbs to achieve additional therapeutic effectiveness. This pharmaceutical approach helps in bringing increased therapeutic effects and reduced adverse effects [1, 2]. Multiple phytoconstituents of a polyherbal formulation may exert synergistic, potentiative, agonist, additive, and antagonistic actions to relieve various disorders [1, 3]. However, herbal preparations are not always safe, as adverse effects were reported on some herbal medicines and polyherbal formulations [4–7]. The presence of extrinsic materials such as heavy metals in some herbal formulations can also cause nephrotoxicity, hepatotoxicity, neurotoxicity, or hematotoxicity. Additionally, interactions with other medicines (herbal or allopathic) also exhibited toxic responses [8, 9].

The growing public interest in herbal therapy, coupled with increasing evidence of the toxic effects of herbal medicines, highlighted the necessity of assessing the toxicity of herbal formulations. There are various approaches to performing toxicological studies of herbal products. In vitro and in vivo studies are the common approaches to evaluate toxicity. The in vivo toxicity studies are considered to be reliable and can be correlated with human outcomes, though ethical and economic issues may arise [10]. Animal toxicity studies can be performed to evaluate various toxicities such as acute toxicity, subacute toxicity, subchronic toxicity, chronic toxicity, reproduction toxicities, carcinogenicity, neurotoxicity, genetic toxicity, and developmental toxicity/embryo toxicities [11]. Acute toxicity studies investigate acute morphological, pathological, or lethal effects of the product in animals [10, 12], while repeated dose toxicity studies, also known as subacute toxicity studies, use relatively low doses to investigate the toxic effect on internal organs. Morphological, histological, and biochemical investigations of the internal organs are performed at the end of the study period [10, 13]. This research work involves acute and subacute toxicity studies of an antiobesity polyherbal formulation, 18KHT01, using male and female ICR mice.

In addition to the toxicological evaluation, polyherbal formulations must undergo quality control assessment to maintain their quality and effectiveness. Quality control ensures that an herbal product has consistent active chemical compositions and biological activity. The increasing utilization of herbal medicine in the present time necessitates assessing the quality of herbal formulations. Therefore, it is essential to develop analytical techniques for quality control and standardization of herbal medicines or polyherbal formulations [14–16]. The chemical constituents present in the polyherbal formulations determine their quality, and their concentrations may vary with different parts of the same plant and be influenced by various environmental and operating factors [16, 17]. Traditionally, the quality of herbal medicine has been assessed by analyzing appearance and odor, but modern techniques involve sensitive analytical methods. Specifically, chemical profiling or chemical fingerprinting is considered a unique and powerful technique for the quality assurance of herbal medicines [17, 18]. Chromatography is a widely used analytical technique for analyzing the chemical fingerprinting of herbal medicines. Though thin-layer chromatography (TLC) is a cheap and rapid technique, high-performance liquid chromatography (HPLC) and ultraperformance liquid chromatography (UPLC) are even more sensitive analytical techniques to analyze herbal products with significant accuracy. Moreover, UPLC coupled with a diode array detector (UPLC-DAD) offers advantages such as high speed, better sensitivity, precision, accuracy, and specificity compared to the HPLC technique [14]. In this study, a rapid and sensitive UPLC-DAD method was developed for the standardization of 18KHT01 formulation, and the method was validated using six marker compounds: caffeine, (-)-epicatechin, corilagin, (-)-epigallocatechin-3-gallate (EGCG), (-)-epicatechin-3-gallate (ECG), and ellagic acid.

18KHT01 is a novel and potent antiobesity composition that showed synergistic antioxidant, antiadipogenic, and antiobesity activities in our previously published study [19]. It potentially inhibited preadipocyte differentiation through the downregulation of vital adipogenic factors such as proliferator-activated receptor gamma (PPAR*γ*), CCAAT enhancer–binding protein alpha (C/EBP*α*), adipocyte protein 2 (aP2), sterol regulatory element–binding transcription factor 1c (SREBP-1c), fatty acid synthase (FAS), and lipoprotein lipase (LPL). The potent antiobesity activity in diet-induced obese mice was achieved through the reduction of major obesity-related parameters such as body weight, weight gain, food efficiency ratio, and serum lipid profile. In addition, 18KHT01 alleviated the high-fat diet–induced diabetes and hepatic steatosis [19]. The objectives of this study are to evaluate broad-spectrum toxicity and validate the analytical method developed for the quality control of 18KHT01 formulation.

## 2. Materials and Methods

### 2.1. Preparation and Extraction of 18KHT01

The method employed for the preparation and extraction of 18KHT01 was described in the previous report [19]. Briefly, *Quercus acutissima* (acorn jelly powder), *Camellia sinensis* (dry leaf buds), *Geranium thunbergii* (dry aerial part), and a small portion of *Citrus limon* (fruit juice) were ground and homogeneously mixed. The formulation was extracted using reflux at 70°C temperature in 40% ethanol solvent. Dry extract was obtained using lyophilization.

### 2.2. Toxicity Study

#### 2.2.1. Experimental Animals

The acute and subacute toxicity study was conducted using ICR mice of either sex. Eight-week-old ICR mice were supplied by Orient Bio (Gyeonggi-do, Korea). The mice were housed under controlled and pathogen-free conditions (room temperature: 24 ± 1°C, relative humidity: 50%–60%, light cycle: 7:00–19:00) and allowed free access to feed and water. Animals were given access to a normal chow diet during the experiment. Animal-related experimental procedures were approved by the Animal Experiment Ethical Committee of Wonkwang University (Approval number: WKU19-76). Animals were handled in accordance with the “Guide for Care and Use of Laboratory Animals” issued by the National Institutes of Health. Toxicity studies were conducted on Good Laboratory Practices in compliance with the Organization for Economic Co-operation and Development (OECD) guidelines [12, 13]. Animals were randomly divided into various groups and acclimatized to the laboratory environment for 1 week before commencement of the experiments. The 18KHT01 extract was suspended into 0.2% carboxymethyl cellulose (CMC) in PBS before being orally administered to mice.

#### 2.2.2. Acute Toxicity Study

The acute oral toxicity of 18KHT01 was conducted as per the guidelines of OECD for oral toxicity study, Section 425 [12]. ICR mice were randomly divided into five groups of either sex. One group was assigned as a control (*n* = 14), and the remaining four groups were treated with 18KHT01 in the doses of 2000 mg/kg (*n* = 10), 2500 mg/kg (*n* = 6), 3000 mg/kg (*n* = 12), and 5000 mg/kg (*n* = 8), respectively. The number of experimental animals mentioned for each group represents the equal number of male and female mice. Animals were fasted overnight prior to dosing, and after 3 h of dosing, they were returned to normal feeding. All the animals were monitored for general behavioral changes, symptoms of toxicity, and mortality on the day of dosing at 1, 3, 5, and 7 h after dosing and thereafter twice daily for 14 days. Animals were monitored for altered autonomic effects (piloerection, salivation, lacrimation, urination, defecation, and rhinorrhoea), central nervous system effects (drowsiness, convulsion, tremors, and sedation), skin ulceration, loss of activity, breathing abnormality, righting reflex, writhing, anxiety, vomiting, diarrhea, and hair loss. The behavioral abnormalities in treatment groups were identified by comparing them with the control group. The body weight, food, and water intake were measured in every 3 days intervals. At the end of the observation period, all animals were sacrificed under anesthesia. The median lethal dose (LD50) and lethal dose (LD100) were calculated by a nonlinear regression procedure [20].

#### 2.2.3. Subacute Oral Toxicity Study

Subacute toxicity study was conducted according to the guidelines of OECD for repeated dose (28 days) oral toxicity study in rodents, Section 407 [13]. Animals were divided into three groups, each having 10 mice (five female and five male). A control group was treated with the vehicle (0.2% CMC/PBS). The remaining two groups were administered with 18KHT01 in a daily dose of 100 mg/kg and 500 mg/kg, respectively, by oral gavage for 30 days. Animals were monitored at least twice daily for behavioral and morphological changes. The mice's body weight, food consumption, and water intake were measured every week.

At the end of the experimental periods (30 days), mice were sacrificed and subjected to necropsy. Before sacrifice, animals were fasted for 16 h. Blood samples were collected from retro-orbital puncture into EDTA (45 mM, pH 7.8) sample tube for hematological analysis and into autoclaved microtubes for serum separation. A complete blood count (CBC) was done using a genus auto hematology analyzer (KT-6200VET, Shenzhen Genius Electronics, China). The serum samples were separated by centrifugation (14,000 rpm for 20 min at 4°C) and used for biochemical analysis. The serum levels of creatinine, blood urea nitrogen (BUN), total bilirubin, and serum alanine aminotransferase (ALT) and aspartate aminotransferase (AST) activities were determined using respective bioassay kits (BioVision, Milpitas, United States) according to the manufacturer's procedure. Visceral organs including liver, kidney, spleen, lungs, heart, genital organs, and brain were harvested, weighed, and stored in 10% formalin/PBS for histopathological analysis. Histopathological analysis was done using hematoxylin and eosin (H&E) staining following the method described in the previous report [21]. The stained slides were examined using an EVOS XL core light microscope (Life Technologies, Bothell, WA, United States), and images were captured at 20× magnification.

### 2.3. UPLC-DAD Method Validation

The procedure for the preparation of the sample and standard solutions for running into the UPLC-DAD and the chromatographic method for the phytochemical analysis were described in the previous report [19]. Briefly, the extracts of the 18KHT01 formulation as well as its herb ingredients were separately dissolved to prepare a 5 mg/mL solution. A mixture of an external standard solution containing caffeine, (-)-epicatechin, corilagin, EGCG, and ECG was prepared, giving the highest stock concentration of 500, 250, 250, 1000, and 500 *μ*g/mL, respectively. Ellagic acid stock solution was separately prepared at a concentration of 300 *μ*g/mL. A dilution of the stock solution of each standard was done for the establishment of a calibration curve. For the phytochemical analysis, (A) acetonitrile and (B) 0.1% phosphoric acid in water were used as chromatographic solvents. The UPLC-DAD analytical method consists of a gradient flow of (A)/(B) = 1/99 (0 min) to (A)/(B) = 20/80 for 35 min with 0.3 mL/min flow rate.

The described analytical method was validated using the validation parameters such as precision, linearity test, limit of detection (LOD), limit of quantification (LOQ), accuracy (recovery) test, and specificity following the guidelines of the International Conference on Harmonization (ICH) [22] and pertinent scientific reports [14, 23–25]. Before performing a method validation, the UPLC system was well-maintained and calibrated. The mobile phase was freshly prepared, and a newly opened Halo 90 Å RP-amide column (Advanced Materials Technology, Munich, Germany) was used.

#### 2.3.1. Linearity, LOD, and LOQ

The stock solutions of external standards were serially diluted to prepare five concentrations. The calibration curves for each marker compound were built by plotting peak areas into the *y*-axis and concentrations (*μ*g/mL) into the *x*-axis. Linearity was appraised by correlation coefficient (*R*^2^). The *R*^2^ value ≥ 0.999 was considered to be linear [23]. The LOD and LOQ were calculated based on the standard deviation (SD) of *y*-intercepts and slopes (*S*) of the calibration curve.

(1) $$\begin{array}{c} \text{LOD}=3.3\times \frac{{\text{SD}}_{\text{Y}-\text{intercept}}}{\text{S}}\\ \text{LOQ}=10\times \frac{{\text{SD}}_{\text{Y}-\text{intercept}}}{\text{S}} \end{array}$$

#### 2.3.2. Precision

The precision of the developed method was determined by assessing repeatability, intraday, and interday variability. The intraday variability was analyzed in five different concentrations of each standard under the optimized condition in a quadruplicate experiment within the same day. Interday variability was carried out by using the same concentrations on three alternative days in the same system. The repeatability test was performed by analyzing the 18KHT01 solution in six sequential experiments. The peak area and retention time for each marker from every repeated experiment were analyzed [24]. All the investigated data were presented in terms of relative standard deviation (RSD). The RSD value ≤ 2% for intraday and ≤ 15% for interday variability was considered to be an acceptable criterion for repeatability precision for the bulk formulation [23, 26].

#### 2.3.3. Specificity

The specificity of the analytical method was evaluated by comparison of peak purity and retention time between marker compounds in the 18KHT01 and that of standards. The UV spectra were compared to confirm the peak purity of standards and marker compounds of 18KHT01 provided by the DAD detector [14].

#### 2.3.4. Accuracy

The recovery tests of the six standards were performed to evaluate the accuracy of the developed analytical method. The 2.5, 5, and 10 *μ*g of each standard were spiked into 5 mg/mL of 18KHT01 solution, and the spiked amount was calculated. The experiments were performed in triplicate, and the accuracy was expressed as measured standard amounts, recovery (%), and RSD (%). The recovery data was calculated using the following equation [14].

(2) $$\begin{array}{c} \text{Recovery }\left(\%\right)=\left(\text{Detected amount}-\text{original amount}\right)\times \frac{100}{\text{Spiked amount}} \end{array}$$

where the original amount is the amount of individual marker compound in 5 mg/mL of 18KHT01, and the detected amount is the total amount in spiked 18KHT01. The spiked amount is the amount of standard added.

#### 2.3.5. Quantification of 18KHT01 and Its Ingredients

The optimized chromatographic method was subsequently applied for the quality evaluation and simultaneous determination of marker compounds in 18KHT01 and individual ingredients. As the lemon was incorporated in the formulation as an olfactory supplement, it was excluded from chromatographic analysis and quantification. The marker compounds were quantified by linear regression using standard curves. Each sample was analyzed three times to determine the mean content of the marker compounds.

### 2.4. Statistical Analysis

The results were presented as mean ± SD. Statistically significant differences between multiple groups in toxicity studies were analyzed with GraphPad Prism 7 software using a one-way analysis of variance (ANOVA) followed by Dunnett's multiple range tests. *p* values less than 0.05 were considered statistically significant.

## 3. Results

### 3.1. Toxicity Study

#### 3.1.1. Clinical Observations and Animal Mortality in Acute Toxicity Study

Mild to severe morbidity symptoms such as piloerection, drowsiness, anxiety, hypoactive, sedation, and laborious breathing were seen in the mice of either sex treated higher than 2500 mg/kg doses of 18KHT01. No sign of morbidity was observed in 2000 mg/kg 18KHT01 treated groups of either sex, indicating 2000 mg/kg as the acute no-observed-adverse-effect level (NOAEL). The clinical symptoms observed in the 2500 mg/kg or higher dose-treated groups were found to be mild to severe, whereas some mild symptoms were relieved gradually. The summary of the clinical observations over the study period was presented in Table S1 (male) and Table S2 (female).

**Figure 1:**
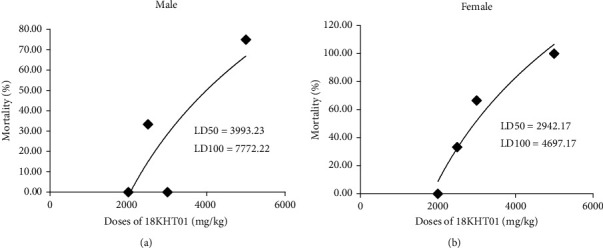
Dose-response mortality curve of acute oral treatment of 18KHT01 in (a) male and (b) female mice. The percentages of mortality values are plotted against the doses of 18KHT01. The LD50 and LD100 values were calculated by interpolation from the logarithmic regression curves.

The mortality induced by different doses of 18KHT01 is presented in Table S3. All the results were plotted into a dose-response nonlinear regression curve as the percentage of mortality versus the administered doses. The LD50 and LD100 were calculated by a nonlinear regression fitting procedure. The LD50 and LD100 for male ICR mice were estimated to be 3993.23 and 7772.22 mg/kg, respectively (Figure 1(a)), whereas those parameters for female mice were estimated to be 2942.17 and 4697.17 mg/kg, respectively (Figure 1(b)).

#### 3.1.2. Effect of 18KHT01 on Food and Water Intake and Body Weight in Subacute Toxicity Study

Food and water intake by control and 18KHT01-treated groups are shown in Figure S1, which were found to be similar among all the experimental groups of the same sex. However, the amounts of food intake by female groups were observed to be lowered than that by male groups. The body weights and weight gains among female groups were not significantly altered, but the 18KHT01 (500 mg/kg)-treated male groups showed significantly decreased body weight after day 8 compared to the male control group (Table S4). Despite similar food intake, the decrease in body weight by the formulation might be because of its antiobesity properties.

#### 3.1.3. Effect of 18KHT01 on Visceral Organ Weights in Subacute Toxicity Study

Mice were sacrificed and various visceral organs including liver, kidneys, spleen, heart, genital organs (testes and ovary), lungs, and brain were isolated, examined for any morphological changes, and measured weight. No gross morphological abnormalities were seen in any of the organs of either sex mice. The weights of the organs are presented in absolute as well as relative weight with fasting body weight (Table 1). No significant differences were observed in the weight of the liver, spleen, lungs heart, genital organs, and brain of 18KHT01 treated male as well as female mice, in comparison to the respective controls. However, the weight of the kidney was found to be significantly decreased in 18KHT01-treated male mice of each dose. Whereas in the formulation-treated female groups, the kidney weight was not significantly altered.

#### 3.1.4. Effect of 18KHT01 on Hematological Parameters in Subacute Toxicity Study

Blood samples were collected from 16 h fasted animals, and a CBC was done on the same day. The hematological analysis results are presented in Table S5. Except for a neutrophil count, which was found to be significantly elevated in 500 mg/kg treated 18KHT01 male mice, no significant differences were observed for other hematological parameters in the 18KHT01-treated mice of either sex.

#### 3.1.5. Effect of 18KHT01 on Liver and Kidney Function Parameters in Subacute Toxicity Study

**Figure 2:**
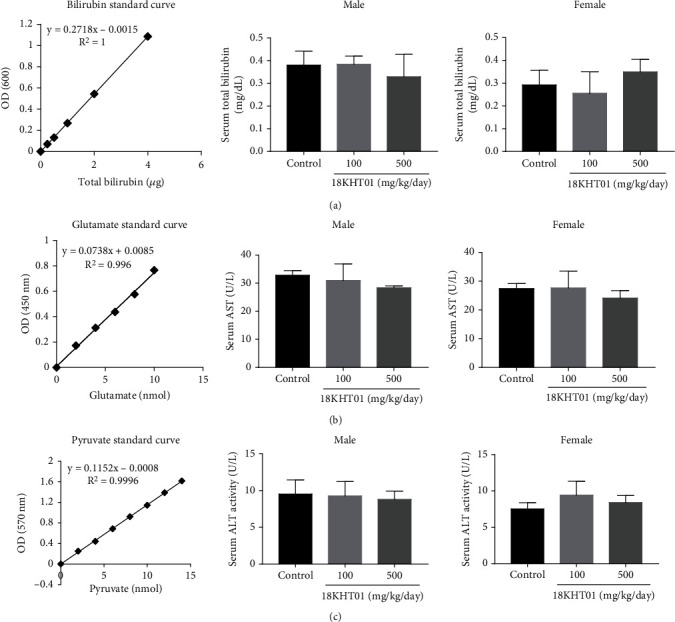
Effect of 18KHT01 on serum (a) total bilirubin level, (b) AST activity, and (c) ALT activity on male and female mice in a 30-day subacute toxicity study. Statistical significance was calculated using one-way ANOVA followed by Dunnett's multiple comparisons test. Results are presented as the mean ± standard deviation (*n* = 5).

**Figure 3:**
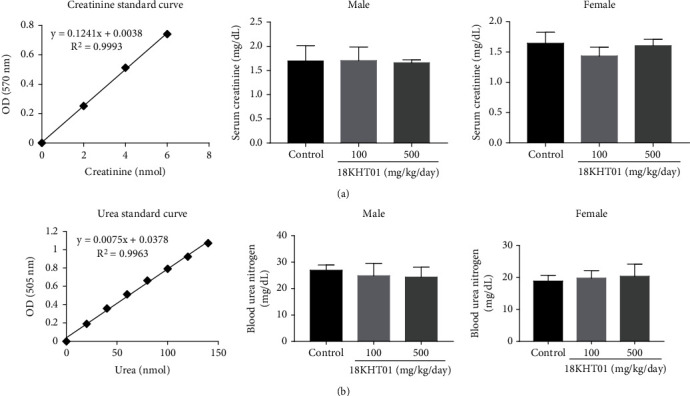
Effect of 18KHT01 on serum (a) creatinine and (b) blood urea nitrogen level on male and female mice in a 30-day subacute toxicity study. Statistical significance was calculated using one-way ANOVA followed by Dunnett's multiple comparisons test. Results are presented as the mean ± standard deviation (*n* = 5).

The liver function test was done by the assessment of AST and ALT activities and total bilirubin levels in serum. As shown in Figure 2, no significant differences were found in the levels of such liver function parameters in any 18KHT01-treated groups in comparison with the respective controls. Similarly, the serum levels of creatinine and urea were analyzed for the assessment of kidney function tests. The serum urea is converted and presented in the form of BUN. As shown in Figure 3, the amounts of such kidney function parameters were not significantly altered in any 18KHT01-treated groups when compared to the respective control.

#### 3.1.6. Effect of 18KHT01 on Histopathology of Visceral Organs in Subacute Toxicity Study

The visceral body organs including the liver, spleen, kidney, heart, and genital organs (testes and ovary) were examined for pathological changes using H&E staining. The histopathological investigations under the light microscope with 20X magnifications did not reveal any treatment-related abnormalities in the male and female mice that received 18KHT01 (Figures S2–S6). In comparison to the control, there was a similar structure and coordination of the cells in each tissue of formulation-treated mice in either sex group.

### 3.2. UPLC-DAD Method Validation

#### 3.2.1. Optimization of Chromatographic Method

**Figure 4:**
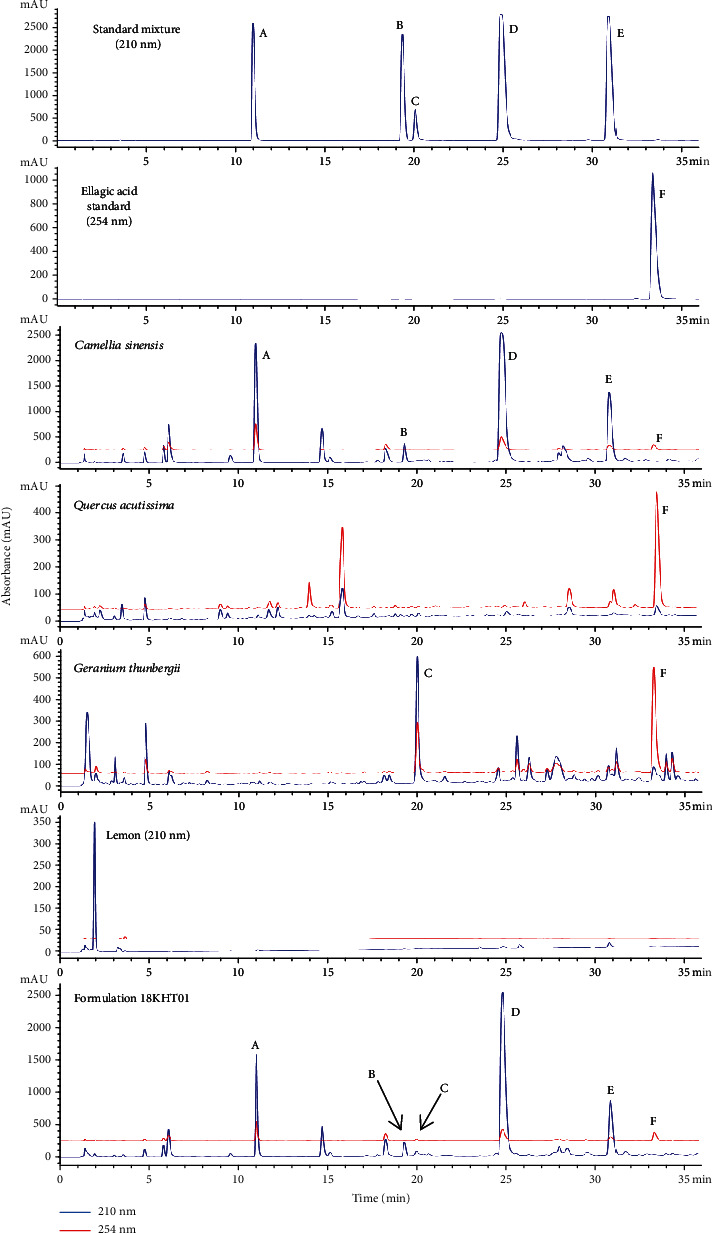
UPLC chromatogram of external standards, individual herb ingredients, and 18KHT01 formulation at 254 nm (red) and 210 nm (blue) [19]. The marker compounds are (A) caffeine; (B) (-)-epicatechin; (C) corilagin; (D) (-)-epigallocatechin-3-gallate (EGCG); (E) (-)-epicatechin-3-gallate (ECG); and (F) ellagic acid.

The UPLC chromatographic method was optimized based on certain conditions such as column type, mobile phase, flow rate, injection volume, running time, and detection wavelength. Various conditions were examined to get a suitable method. For 18KHT01, the best condition was optimized as follows: column type: Halo 90 Å RP-amide column (2 *μ*m particle sizes, 2.1 × 150 mm); column temperature: 40°C; mobile phase: acetonitrile (A) and 0.1% phosphoric acid in water (B); elution system: gradient flow (*A*)/(*B*) = 1/99 (0 min)➔(*A*)/(*B*) = 20/80 (35 min); flow rate: 0.3 mL/min; detection wavelength: 210 nm and 254 nm; and injection volume: 2 *μ*L. As presented in the previous report [19], the typical UPLC chromatograms of the 18KHT01 formulation, external standard mixture, and individual herb ingredients obtained by running with the optimized chromatographic method are shown in Figure 4.

#### 3.2.2. Linearity, LOD, and LOQ

The linearity of the developed method was determined by plotting five different concentrations and the peak area of each external standard. The correlation coefficient (*R*^2^) values of all standard calibration curves were more than 0.9997 showing good linearity in the injected ranges. The LOD is the lowest concentration of analyte in a solution that can be detectable, but not necessarily be quantified, while the LOQ is the lowest concentration of analyte in a solution that can be quantified with acceptable precision and accuracy under the stated methods [14]. The LOD and LOQ were shown to have values in the range of 1.89~11.456 and 5.73~34.72 *μ*g/mL, respectively, for the standard compounds. The regression equation, correlation coefficients (*R*^2^), SD of *Y*-intercepts, LOD, and LOQ values for each standard compound are presented in Table 2.

#### 3.2.3. Precision

The analyzed data showed that the RSDs of repeatability of the detected amount of the markers in 18KHT01 were observed in the range of 0.71%~1.13%, and the RSDs of repeatability of retention time of these markers were in the range of 0.07%~0.19%, which were in acceptable criteria (Table 3). Moreover, the RSD of intraday and interday variability for analytical standards were, respectively, in the range of 0.20%~1.75% and 0.16%~3.93% (Table 4). None of the precision data exceeded 5%, indicating that the RSD values of each external standard for intraday and interday analysis were in an acceptable range. These data revealed that the described method maintained an acceptable degree of precision.

#### 3.2.4. Specificity

**Figure 5:**
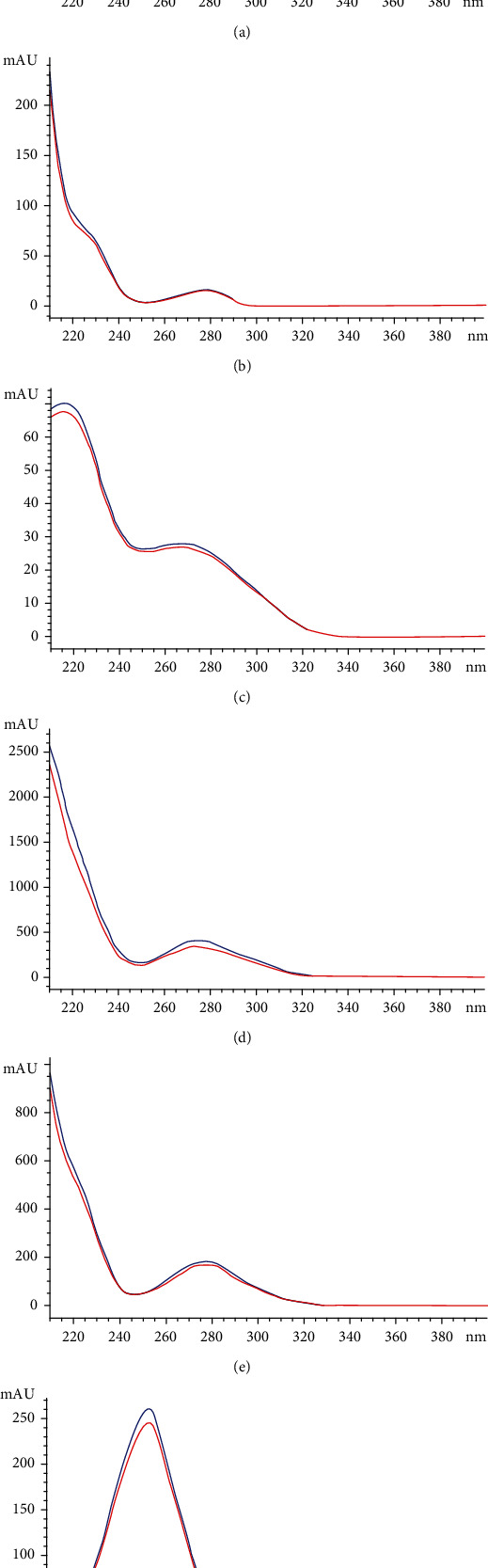
UV spectra of six marker compounds: (a) caffeine; (b) (-)-epicatechin; (c) corilagin; (d) (-)-epigallocatechin-3-gallate (EGCG); (e) (-)-epicatechin-3-gallate (ECG), and (f) ellagic acid. The blue curves represent the UV spectra of marker compounds assigned as standard, while the red curves represent the UV spectrum of the marker compound in 18KHT01.

The patterns of UV absorption between standard compounds and marker compounds of 18KHT01 were compared to evaluate peak purity. Standards and samples were measured at the same wavelength, and no interferences were observed due to impurities (Figure 5). The retention time of standards (*n* = 4) and markers of 18KHT01 (*n* = 6) were also compared and were observed to be similar enough (Table S6).

#### 3.2.5. Accuracy

Accuracy assay was performed by recovery test. The result showed that the percentage of recovery of standards was found to be in the range of 95.89%~103.28%, and RSD values were in the range of 0.86%~4.42% (Table 5) which was in an acceptable range. The recovery data represented the accuracy of the developed method and is sufficient for the usual analysis of 18KHT01.

#### 3.2.6. Quality Assurance of 18KHT01 and Its Ingredients

All the herb ingredients, as well as the 18KHT01 formulation, were individually analyzed using the optimized validated chromatographic method to quantify the marker compounds. The content of these marker compounds in both 18KHT01 and its ingredients is presented in Table 6.

## 4. Discussion

World Health Organization (WHO) reports, along with other publications, stated that more than 80% (and still in increasing outline) of the world's population, especially in the developing world, depends on the herbal and traditional systems of medicines as a first line of therapy for healing [4, 27, 28]. However, there are ongoing concerns about the safety and effectiveness of such herbal therapies. Contrary to popular belief, herbal medicines are not always safe. Large doses of herbal products can lead to severe, life-threatening effects and even death [4]. Therefore, it is mandatory to perform a toxicological evaluation before marketing any herbal formulations. In this study, we conducted acute and 30-day repeated dose subacute toxicological studies on an antiobesity polyherbal formulation, 18KHT01.

In the acute toxicity study, mild to severe treatment-related clinical signs such as drowsiness, piloerection, anxiety, hypoactive, sedation, and/or labored breathing were observed in the mice treated with doses above 2500 mg/kg of 18KHT01. Similar signs of toxicity have been reported in previous studies with EGCG-treated mice and rats [29, 30]. As EGCG is the major compound in 18KHT01 (Table 6), the observed toxicity may be attributed to its presence. Previous studies on *C. sinensis*, a major component of 18KHT01, have shown severe adverse symptoms including lethargy, abnormal breathing, ataxia, and even death in mice at high acute doses (1000 mg/kg), with liver necrosis identified as a major underlying cause of death [31]. Tannins from QA have also shown renal toxicity in ruminant animals, leading to severe nephrosis and death in cattle [32, 33]. Clarke and Cotchin found that hydrolysable tannins from acorns were responsible for mortality in mice, rabbits, and calves when administered via injectable routes [34]. Therefore, it is plausible that hepatic and renal disorders could be the underlying cause of mortality observed at higher acute doses of 18KHT01. The dose-response nonlinear regression curve between the percentage of mortality and the administered doses revealed that the LD50 of 18KHT01 was 3993.23 mg/kg for male mice and 2942.17 mg/kg for female mice (Figure 1), suggesting that female mice are more susceptible to 18KHT01 than males. Similarly, EGCG has shown higher toxicity levels in female Wistar rats compared to males [30].

Following the acute toxicity testing, a 30-day repeated dose toxicity study was conducted to investigate the toxic responses of 18KHT01 on various targets and body systems. During this period, male mice treated with 500 mg/kg of 18KHT01 showed a significant reduction in body weight (Table S4). Green tea polyphenols and catechins are known to alter intestinal food absorption, and tannins interact with food nutrients, reducing their nutritional value [35–37]. The high levels of polyphenols, catechins, and tannins in 18KHT01 might decrease food absorption, thereby altering body weight despite similar food intake in the formulation-treated groups. The reduced body weight might have influenced kidney weights in the 18KHT01-treated male group (Table 1). Previous studies have shown that kidney weight is directly correlated with body weight in mice, but not with the number of nephrons [38, 39]. No kidney toxicity was supported by serum creatinine and BUN assessments, which revealed no significant differences among the experimental groups of the same sex (Figure 3). Previous studies have demonstrated that green tea, a major component of 18KHT01, shows renal protective action by significantly decreasing creatinine and BUN levels [40–42].

Liver function tests revealed that serum total bilirubin (unconjugated plus conjugated) levels and ALT and AST activities in 18KHT01-treated groups were not significantly different from the respective controls, indicating that 18KHT01 is safe for the liver in ICR mice at doses below 500 mg/kg. Additionally, UPLC standardization of 18KHT01 revealed that it contained 155.70 ± 1.35 mg/g of EGCG (Table 6), equating to an intake of about 77.5 mg EGCG/kg during ingestion of 500 mg/kg of 18KHT01. Previous studies have shown that 67.8 and 108 mg/kg oral doses of pure EGCG exhibit liver toxicity with fatty changes [43]. The lack of adverse effects on the liver in this study suggests that the adverse effects of EGCG may be mitigated by the negative synergistic action of multiple components in 18KHT01.

Toxic chemicals may cause hematological disorders by integration with the blood constituents [44, 45]. Hematological evaluations are conducted to diagnose adverse effects on blood parameters. In this study, a slight increase in neutrophil count was noted in mice treated with 500 mg/kg of 18KHT01. These alterations in neutrophil count were not considered toxicologically significant as they were limited to one sex, not dose-dependent, and not supported by changes in other blood parameters. Overall, the toxicity study results suggest that acute doses below 2000 mg/kg and subacute doses below 500 mg/kg of 18KHT01 are safe for male and female ICR mice.

In addition to toxicity, product quality is another issue for the utilization of herbal medicines in a health care system. Various factors, such as geographical location, climate conditions, environmental hazards, harvesting methods, and collecting protocols, affect the phytochemical variation in plants and so affect the quality of herbal medicine [1]. The chemical profile reflects the quality of any herbal medicine, determining the safety and efficacy [46]. Various analytical methods are used to analyze the chemical profile, and validation of these methods is essential for reproducible results. In this research, a UPLC-DAD method was developed for the simultaneous analysis of the 18KHT01 polyherbal formulation and validated according to the ICH guidelines and scientific literature [14, 22–26]. The results showed that parameters such as linearity, precision, specificity, and accuracy were within the acceptance criteria for the developed method.

18KHT01 consists of three active ingredient herbs: *C. sinensis*, *Q. acutissima*, and *G. thunbergii*, with a small amount of *C. limon* (lemon) as an olfactory supplement. Unfermented green leaf buds, known as green tea, were selected as the major portion of 18KHT01. Catechins (flavan-3-ols) constitute 30%–40% of the total compounds in green tea, with major components including catechin, epicatechin, epigallocatechin (EGC), ECG, EGCG, and gallocatechin gallate [47]. EGCG is the most abundant catechin derivative in green tea, comprising 50%–80% of total catechins [48, 49]. This study confirmed EGCG as a major component of *C. sinensis* and 18KHT01 (Table 6). Similarly, ellagitannins, including corilagin and geraniin, are major constituents of *G. thunbergia* [50]. Thus, corilagin was selected as one of the marker compounds for the analysis of 18KHT01. The 40% ethanol extract of *G. thunbergia* and 18KHT01 was composed of 35.76 ± 0.14 mg/g and 6.07 ± 0.06 mg/g of corilagin, respectively (Table 6). Ellagic acid was observed to be a major component in the ethanol extract of *Q. acutissima*. Thus, ellagic acid was selected as one of the marker compounds for analysis of *Q. acutissima* and 18KHT01.

In general, all the marker compounds were detected and determined in 18KHT01, with EGCG being the most abundant, followed by caffeine, ECG, ellagic acid, corilagin, and epicatechin. Each validation parameter, including linearity, precision, specificity, and accuracy, met the acceptance criteria, indicating that the developed method is simple, accurate, sensitive, precise, specific, and reproducible for simultaneous analysis and quantification of the marker compounds in 18KHT01.

This study concluded that the polyherbal formulation 18KHT01 is safe for chronic oral administration in mice at doses below 500 mg/kg/day and has a NOAEL of 2000 mg/kg for acute administration, with higher susceptibility observed in female mice. The analytical and method validation study provided a validated UPLC-DAD chromatographic method with acceptable levels of linearity, precision, specificity, and accuracy for the quality control and standardization of the 18KHT01 formulation. Future research should explore the mechanisms of observed toxicological effects, conduct long-term studies to assess chronic toxicity and potential carcinogenicity, and eventually proceed to clinical trials in humans.

## Figures and Tables

**Table 1:** Effect of 18KHT01 on weights of vital organs of male and female mice in 30 days subacute toxicity study.

**Organs**	**Male**			**Female**		
	**Control**	**18KHT01 (mg/kg)**		**Control**	**18KHT01 (mg/kg)**	
		**100**	**500**		**100**	**500**
Fasting body weight (g)	41.31 ± 2.06	39.55 ± 2.42	37.76 ± 2.95	31.87 ± 0.28	33.12 ± 2.2	31.31 ± 3.7
Liver (g)	1.63 ± 0.18	1.41 ± 0.11	1.37 ± 0.22	1.20 ± 0.12	1.34 ± 0.14	1.13 ± 0.27
Liver (% of body weight)	3.95 ± 0.37	3.56 ± 0.13	3.61 ± 0.37	3.76 ± 0.34	4.04 ± 0.24	3.60 ± 0.62
Kidney (g)	0.65 ± 0.03	0.51 ± 0.1^∗∗^	0.52 ± 0.07^∗∗^	0.37 ± 0.05	0.37 ± 0.03	0.40 ± 0.04
Kidney (% of body weight)	1.57 ± 0.10	1.28 ± 0.1^∗∗^	1.37 ± 0.14^∗^	1.16 ± 0.14	1.12 ± 0.06	1.29 ± 0.04
Spleen (g)	0.10 ± 0.02	0.11 ± 0.02	0.10 ± 0.02	0.13 ± 0.02	0.17 ± 0.04	0.13 ± 0.03
Spleen (% of body weight)	0.25 ± 0.05	0.26 ± 0.03	0.26 ± 0.05	0.39 ± 0.07	0.50 ± 0.10	0.42 ± 0.08
Heart (g)	0.23 ± 0.02	0.19 ± 0.03	0.18 ± 0.03^∗^	0.16 ± 0.03	0.20 ± 0.03	0.19 ± 0.03
Heart (% of body weight)	0.55 ± 0.07	0.47 ± 0.07	0.47 ± 0.04	0.50 ± 0.08	0.61 ± 0.07	0.59 ± 0.07
Lungs (g)	0.23 ± 0.03	0.21 ± 0.01	0.21 ± 0.01	0.20 ± 0.02	0.22 ± 0.02	0.24 ± 0.05
Lungs (% of body weight)	0.56 ± 0.08	0.53 ± 0.06	0.56 ± 0.06	0.62 ± 0.06	0.68 ± 0.07	0.77 ± 0.1^∗^
Testes (g)	0.27 ± 0.03	0.25 ± 0.03	0.25 ± 0.06	—	—	—
Testes (% of body weight)	0.65 ± 0.07	0.63 ± 0.07	0.66 ± 0.13	—	—	—
Ovary (g)	—	—	—	41.43 ± 15.45	39.58 ± 9.1	34.26 ± 6.5
Ovary (% of body weight)	—	—	—	0.13 ± 0.05	0.12 ± 0.04	0.11 ± 0.03
Brain (g)	0.49 ± 0.02	0.49 ± 0.02	0.48 ± 0.02	0.49 ± 0.01	0.49 ± 0.01	0.49 ± 0.05
Brain (% of body weight)	1.18 ± 0.09	1.23 ± 0.09	1.27 ± 0.11	1.54 ± 0.03	1.48 ± 0.09	1.58 ± 0.16

*Note:* Statistical significance was calculated using one-way ANOVA followed by Dunnett's multiple comparisons test. Results are presented as the mean ± standard deviation (*n* = 5) with significance.

^∗^ *p* < 0.05 vs. controls of the respective sex group.

^∗∗^ *p* < 0.01 vs. controls of the respective sex group.

**Table 2:** UPLC analyzed regression data, LODs, and LOQs for marker compounds (*n* = 4).

**Markers**	**Regression equation**	**R** ^2^	**S** **D**_**Y**−**intercept**_	**LOD**	**LOQ**
Caffeine	*y* = 39.99*x* + 566.94	0.9997	104.31	8.607431	26.08312
Epicatechin	*y* = 88.47*x* + 518.53	0.9998	86.13	3.212644	9.735285
Corilagin	*y* = 22.076*x* + 51.272	0.9999	12.65	1.89047	5.728697
EGCG	*y* = 54.437*x* + 1858.7	0.9999	188.98	11.45625	34.71589
ECG	*y* = 89.785*x* + 1021.7	0.9999	101.28	3.722341	11.27982
Ellagic acid	*y* = 45.018*x* + 78.392	0.9999	44.46	3.258874	9.875376

*Note:y*: peak area; *x*: sample concentration (*μ*g/mL); *R*^2^: correlation coefficients.

Abbreviations: LOD, limit of detection; LOQ, limit of quantification.

**Table 3:** Repeatability of marker compounds of 18KHT01.

**Markers**	**Amount (*μ*g/mL)** **Mean ± SD (** **n** ** = 6)**	**RSD (%)**	**Retention time (min)** **Mean ± SD (** **n** ** = 6)**	**RSD (%)**
Caffeine	274.45 ± 2.14	0.78	10.69 ± 0.02	0.19
Epicatechin	16.61 ± 0.19	1.13	19.19 ± 0.03	0.13
Corilagin	30.35 ± 0.29	0.95	19.92 ± 0.02	0.10
EGCG	778.50 ± 6.77	0.87	24.93 ± 0.02	0.08
ECG	122.52 ± 1.06	0.86	31.09 ± 0.02	0.07
Ellagic acid	61.30 ± 0.43	0.71	33.28 ± 0.02	0.07

Abbreviations: RSD, relative standard deviation; SD, standard deviation.

**Table 4:** Analytical results of intraday and interday variability of 18KHT01.

**Marker compounds**	**Concentrations (*μ*g/mL)**	**Intraday variability (** **n** ** = 6)**		**Interday variability (** **n** ** = 6)**	
		**Mean ± SD (** ***μ*** **g/mL)**	**RSD (%)**	**Mean ± SD (** ***μ*** **g/mL)**	**RSD (%)**
Caffeine	500	498.44 ± 4.05	0.81	497.45 ± 15.8	3.17
	250	251.42 ± 1.19	0.47	253.13 ± 2.86	1.13
	125	130.17 ± 1.31	1.00	130.53 ± 2.46	1.89
	62.5	60.59 ± 0.13	0.21	62.23 ± 1.84	2.96
	31.25	28.14 ± 0.28	1.01	25.41 ± 0.80	3.14

Epicatechin	250	249.33 ± 2.20	0.55	248.83 ± 6.45	2.59
	125	125.75 ± 0.97	0.77	126.73 ± 1.88	1.49
	62.5	64.31 ± 0.71	1.10	64.33 ± 1.55	2.41
	31.25	30.55 ± 0.06	0.21	31.23 ± 0.21	0.66
	15.625	14.43 ± 0.20	1.39	13.26 ± 0.52	3.93

Corilagin	250	250.25 ± 2.42	0.97	250.07 ± 6.73	2.69
	125	124.92 ± 1.70	1.36	124.32 ± 1.50	1.21
	62.5	61.34 ± 0.69	1.12	63.46 ± 1.26	1.98
	31.25	31.38 ± 0.47	1.50	32.10 ± 1.21	3.76
	15.625	16.47 ± 0.29	1.75	14.42 ± 0.54	3.75

EGCG	1000	1002.35 ± 8.03	0.80	997.73 ± 5.65	0.57
	500	493.49 ± 1.99	0.40	498.49 ± 2.44	0.49
	250	251.85 ± 0.88	0.35	264.08 ± 0.41	0.16
	125	129.68 ± 0.38	0.29	127.21 ± 2.20	1.73
	62.5	60.13 ± 0.75	1.25	49.97 ± 0.39	0.79

ECG	500	499.09 ± 3.35	0.67	498.06 ± 8.52	1.71
	250	252.12 ± 2.85	1.13	252.76 ± 1.95	0.77
	125	124.35 ± 0.49	0.39	127.85 ± 0.89	0.70
	62.5	63.33 ± 0.10	0.16	63.79 ± 0.62	0.97
	31.25	29.87 ± 0.14	0.48	26.30 ± 0.22	0.85

Ellagic acid	300	299.05 ± 1.63	0.55	298.39 ± 5.22	1.75
	150	151.73 ± 0.50	0.33	153.34 ± 5.14	3.35
	75	75.96 ± 0.15	0.20	75.57 ± 1.99	2.64
	37.5	36.77 ± 0.27	0.75	36.55 ± 1.25	3.43
	18.75	17.74 ± 0.05	0.28	17.39 ± 0.59	3.39

Abbreviations: RSD, relative standard deviation; SD, standard deviation.

**Table 5:** Recovery of each marker compound added into 18KHT01 solution.

**Markers**	**Spiked amount (*μ*g)**	**Measured amount (*μ*g)** **Mean ± SD** **n** ** = 3)**	**Recovery (%)**	**RSD (%)**
Caffeine	10	10.03 ± 0.24	100.03	2.36
	5	5.09 ± 0.12	103.28	2.32
	2.5	2.50 ± 0.10	98.22	4.04

Epicatechin	10	10.08 ± 0.10	100.86	1.00
	5	4.92 ± 0.08	98.20	1.65
	2.5	2.47 ± 0.02	99.11	1.01

Corilagin	10	10.11 ± 0.33	100.90	3.22
	5	5.01 ± 0.05	101.19	0.95
	2.5	2.52 ± 0.11	99.24	4.19

EGCG	10	10.10 ± 0.25	100.81	2.43
	5	5.07 ± 0.36	102.40	7.01
	2.5	2.47 ± 0.02	97.20	0.86

ECG	10	10.02 ± 0.13	100.41	1.32
	5	4.94 ± 0.15	97.51	2.95
	2.5	2.48 ± 0.11	100.82	4.42

Ellagic acid	10	9.99 ± 0.09	99.78	0.91
	5	4.94 ± 0.06	99.61	1.28
	2.5	2.44 ± 0.05	95.89	2.19

*Note:* The recovery data was calculated using the following equation: Recovery (%) = (Detected amount–original amount) × 100/Spiked amount.

Abbreviations: RSD, relative standard deviation; SD, standard deviation.

**Table 6:** Content of marker compounds in 18KHT01 and individual herbs used in the formulation.

**Samples**	**Markers**	**Amount (mg/g)** **Mean ± SD**
18KHT01	Ellagic acid	12.26 ± 0.086
	Caffeine	54.89 ± 0.43
	Epicatechin	3.32 ± 0.04
	Corilagin	6.07 ± 0.06
	EGCG	155.70 ± 1.35
	ECG	24.50 ± 0.21

*Camellia sinensis*	Ellagic acid	4.93 ± 0.12
	Caffeine	87.44 ± 0.21
	Epicatechin	6.07 ± 0.03
	EGCG	213.43 ± 0.59
	ECG	39.21 ± 0.17

*Geranium thunbergii*	Ellagic acid	21.53 ± 0.05
	Corilagin	35.76 ± 0.14

*Quercus acutissima*	Ellagic acid	20.07 ± 0.09

*Note:* Data are presented as mean ± SD (*n* = 3 for herbs; *n* = 6 for 18KHT01).

## Data Availability

The data associated with this study are available from the corresponding author upon reasonable request.

## Supporting Information

Supporting Information Additional supporting information can be found online in the Supporting Information section. A summary of clinical observation, food and water intake, body weight variation, hematological parameters, and mortality of the experimental mice, as well as histological changes in the liver, spleen, kidney, heart, and genital organs, is presented in the supporting information.
