# Supplementary material for: Toxicological Evaluation of a Polyherbal Formulation (18KHT01) and Validation of UPLC-DAD Method for Quality Control
Source: Biomed Res Int. 2024 Sep 19;2024:1767618. doi: 10.1155/2024/1767618 (PMC11427720; doi:10.1155/2024/1767618)
Supplement: Supporting Information — Additional supporting information can be found online in the Supporting Information section. A summary of clinical observation, food and water intake, body weight variation, hematological parameters, and mortality of the experimental mice, as well as histological changes in the liver, spleen, kidney, heart, and genital organs, is presented in the supporting information. [file 1767618.f1.docx]

Supplementary Material

**Toxicological evaluation of a Polyherbal Formulation (18KHT01) and Validation of UPLC-DAD Method for Quality Control**

Prakash Raj Pandeya^1,2^, Ramakanta Lamichhane^3^, Gopal Lamichhane^1,4^, Kyung-Hee Lee^1^, and Hyun-Ju Jung^1^*

^1^Department of Oriental Pharmacy and Wonkwang-Oriental Medicines Research Institute, Wonkwang University, Sinyong-Dong, Iksan 570-749, South Korea

^2^Department of Animal and Food Sciences, University of Kentucky, Lexington, KY 40546, USA

^3^Department of Pharmacy, Kathmandu University, Dhulikhel 45200, Nepal

^4^Department of Nutritional Sciences, Oklahoma State University, Stillwater, OK 74078, USA

* Correspondence: [hyun104@wku.ac.kr](mailto:hyun104@wku.ac.kr) (HJJ)

Author’s e-mail addresses:

[pandeya.praj@gmail.com](mailto:pandeya.praj@gmail.com) (PRP)

[ramakanta.lamichhane@ku.edu.np](mailto:ramakanta.lamichhane@ku.edu.np) (RL)

[lamichhanegopal1@gmail.com](mailto:lamichhanegopal1@gmail.com) (GL)

[fuhaha112@naver.com](mailto:fuhaha112@naver.com) (KHL)

**Supplemental Tables**

TABLE S1: Summary of clinical observations in male mice in acute toxicity studies.

| **Doses** | **Days** | **Observed**  **frequency** | | **Signs of toxicity- Male** | | | | | |
| --- | --- | --- | --- | --- | --- | --- | --- | --- | --- |
|  |  |  |  | **Piloerection** | **Drowsiness** | **Sedation** | **Labored breathing** | | **Death** |
| **2000 mg/kg**  **(n=5)** | Day 1 | 1^st^ | | - | - | - | - | | - |
|  |  | 2^nd^ | | - | - | - | - | | - |
|  |  | 3^rd^ | | - | - | - | - | | - |
|  |  | 4^th^ | | - | - | - | - | | - |
|  | Day 2-14 | | | - | - | - | - | | - |
|  | | | | | | | | | |
| **2500 mg/kg**  **(n=3)** | Day 1 | | 1^st^ | - | 2/3 | - | - | - | |
|  |  |  | 2^nd^ | - | - | - | - | - | |
|  |  |  | 3^rd^ | - | - | - | - | - | |
|  |  |  | 4^th^ | - | 1/3 | - | - | - | |
|  | Day 2 | | 1^st^ | 1/3 | 1/3 | - | - | - | |
|  |  |  | 2^nd^ | 2/3 | 1/3 | - | 1/3 | - | |
|  | Day 3 | | 1^st^ | - | - | - | - | 1/3 | |
|  |  |  | 2^nd^ | - | - | - | - | - | |
|  | Day 4 | | 1^st^ | 1/2 | - | - | - | - | |
|  |  |  | 2^nd^ | 1/2 | - | - | - | - | |
|  | Day 5-14 | | | - | - | - | - | - | |
|  | | | | | | | | | |
| **3000 mg/kg**  **(n=6)** | Day 1 | | 1^st^ | - | - | - | - | - | |
|  |  |  | 2^nd^ | - | - | - | - | - | |
|  |  |  | 3^rd^ | - | 1/6 | - | - | - | |
|  |  |  | 4^th^ | - | 1/6 | - | - | - | |
|  | Day 2 | | 1^st^ | - | - | - | - | - | |
|  |  |  | 2^nd^ | 1/6 | 1/6 | - | - | - | |
|  | Day 3 | | 1^st^ | 1/6 | - | - | - | - | |
|  |  |  | 2^nd^ | 1/6 | 1/6 | - | - | - | |
|  | Day 4-14 | | | - | - | - | - | - | |
|  | | | | | | | | | |
| **5000 mg/kg**  **(n=4)** | Day 1 | | 1^st^ | 2/4 | 1/4 | - | - | - | |
|  |  |  | 2^nd^ | 3/4 | 2/4 | - | - | - | |
|  |  |  | 3^rd^ | 2/4 | 2/4 | - | - | - | |
|  |  |  | 4^th^ | 2/4 | 2/4 | - | - | - | |
|  | Day 2 | | 1^st^ | 2/4 | 1/4 | - | - | - | |
|  |  |  | 2^nd^ | 3/4 | 1/4 | - | - | - | |
|  | Day 3 | | 1^st^ | 4/4 | 4/4 | - | - | - | |
|  |  |  | 2^nd^ | 4/4 | 3/4 | 1/4 | 3/4 | - | |
|  | Day 4 | | 1^st^ | 2/4 | 1/4 | - | 1/4 | 2/4 | |
|  |  |  | 2^nd^ | 2/2 | - | 1/2 | 1/2 | - | |
|  | Day 5 | | | 1/2 | 1/2 | - | - | 1/2 | |
|  | Day 6-14 | | | - | - | - | - | - | |

̶ : absent of the clinical symptoms

TABLE S2: Summary of clinical observations in female mice in acute toxicity studies.

| **Doses** | **Days** | | **Observed**  **frequency** | | **Signs of toxicity- Female** | | | | |
| --- | --- | --- | --- | --- | --- | --- | --- | --- | --- |
|  |  |  |  |  | **Piloerection** | **Drowsiness** | **Sedation** | **Labored breathing** | **Death** |
| **2000 mg/kg**  **(n=5)** | Da 1 | | 1^st^ | | - | - | - | - | - |
|  |  |  | 2^nd^ | | - | - | - | - | - |
|  |  |  | 3^rd^ | | - | - | - | - | - |
|  |  |  | 4^th^ | | - | - | - | - | - |
|  | Day 2-14 | | | | - | - | - | - | - |
|  | | | | | | | | | |
| **2500 mg/kg**  **(n=3)** | | Day 1 | | 1^st^ | - | 1/3 | - | - | - |
|  |  |  |  | 2^nd^ | - | 1/3 | - | - | - |
|  |  |  |  | 3^rd^ | - | - | - | - | - |
|  |  |  |  | 4^th^ | - | 1/3 | - | - | - |
|  |  | Day 2 | | 1^st^ | 1/3 | 1/3 | - | - | - |
|  |  |  |  | 2^nd^ | 1/3 | 1/3 | 1/3 | - | - |
|  |  | Day 3 | | 1^st^ | - | - | - | - | 1/3 |
|  |  |  |  | 2^nd^ | - | - | - | - | - |
|  |  | Day 4-14 | | | - | - | - | - | - |
|  | | | | | | | | | |
| **3000 mg/kg**  **(n=6)** | | Day 1 | | 1^st^ | - | - | - | - | - |
|  |  |  |  | 2^nd^ | - | - | - | - | - |
|  |  |  |  | 3^rd^ | - | 1/6 | - | - | - |
|  |  |  |  | 4^th^ | 1/6 | 1/6 | - | - | - |
|  |  | Day 2 | | 1^st^ | 1/6 | 1/6 | - | 1/6 | - |
|  |  |  |  | 2^nd^ | 2/6 | 1/6 | 1/6 | 2/6 | 1/6 |
|  |  | Day 3 | | 1^st^ | - | - | - | - | 3/5 |
|  |  |  |  | 2^nd^ | - | - | - | - | - |
|  |  | Day 4-14 | | | - | - | - | - | - |
|  | | | | | | | | | |
| **5000 mg/kg**  **(n=4)** | | Day 1 | | 1^st^ | 2/4 | - | - | - | - |
|  |  |  |  | 2^nd^ | 1/4 | 1/4 | 1/4 | 1/4 | - |
|  |  |  |  | 3^rd^ | 2/4 | 1/4 | - | 1/4 | - |
|  |  |  |  | 4^th^ | 1/4 | - | 1/4 | 1/4 | - |
|  |  | Day 2 | | 1^st^ | 3/4 | - | 1/4 | - | 1/4 |
|  |  |  |  | 2^nd^ | 3/3 | - | 3/3 | 1/3 | - |
|  |  | Day 3 | | 1^st^ | - | - | - | - | 3/3 |

̶ : absent of the clinical symptoms

TABLE S3: Mortality and survival of ICR mice by acute oral treatment of 18KHT01.

| **Groups** | **Total number of animals** | **Number of animals died** | **Number of animals survived** | **Mortality (%)** | **Survival (%)** |
| --- | --- | --- | --- | --- | --- |
| **Male** | | | | | |
| Control | 7 | 0 | 7 | 0.00 | 100.00 |
| 2000 mg/kg | 5 | 0 | 5 | 0.00 | 100.00 |
| 2500 mg/kg | 3 | 1 | 2 | 33.33 | 66.67 |
| 3000 mg/kg | 6 | 0 | 6 | 0.00 | 100.00 |
| 5000 mg/kg | 4 | 3 | 1 | 75.00 | 25.00 |
| **Female** | | | | | |
| Control | 7 | 0 | 7 | 0.00 | 100.00 |
| 2000 mg/kg | 5 | 0 | 5 | 0.00 | 100.00 |
| 2500 mg/kg | 3 | 1 | 2 | 33.33 | 66.67 |
| 3000 mg/kg | 6 | 4 | 2 | 66.67 | 33.33 |
| 5000 mg/kg | 4 | 4 | 0 | 100.00 | 0.00 |

TABLE S4: Body weight variation and weight gain on control and 18KHT01 treated male and female mice in 30 days sub-acute toxicity study.

| **Groups** | **Day 0** | **Day 8** | **Day 14** | **Day 22** | **Day 30** | **Weight gain** | | |
| --- | --- | --- | --- | --- | --- | --- | --- | --- |
| **Male** | | | | | | | |  |
| Control | 41.54 ± 2.3 | 41.85 ± 2.1 | 42.75 ± 2.5 | 42.66 ± 1.3 | 44.58 ± 2.1 | | 3.04 ± 1.7 |  |
| 100 mg/kg | 40.35 ± 1.5 | 39.80 ± 1.2 | 40.01 ± 1.8 | 40.1 ± 1.5* | 41.38 ± 2.1 | | 1.03 ± 1.6 |  |
| 500 mg/kg | 39.61 ± 1.2 | 38.9 ± 1.1* | 38.5 ± 1.8* | 39.3 ±1.8** | 40.4 ± 3.1* | | 0.77 ± 3.1 |  |
| **Female** | | | | | | | |  |
| Control | 32.32 ± 1.4 | 32.46 ± 1.7 | 32.98 ± 1.1 | 33.28 ± 1.2 | 35.16 ± 0.6 | | 2.83 ± 1.1 |  |
| 100 mg/kg | 33.71 ± 2.8 | 32.65 ± 1.4 | 33.77 ± 2.2 | 32.99 ± 1.3 | 36.05 ± 2.7 | | 2.33 ± 1.0 |  |
| 500 mg/kg | 32.26 ± 1.6 | 31.22 ± 1.3 | 31.14 ± 2.2 | 31.57 ± 1.6 | 34.17 ± 4.2 | | 1.91 ± 2.8 |  |

Statistical significance was calculated using one-way ANOVA followed by Dunnett's multiple comparisons test. Results are presented as the mean ± standard deviation (n=5) with significance **P*<0.05, ***P*<0.01 vs. controls of respective sex group.

TABLE S5: Effect of 18KHT01 on hematological parameters of male and female mice in 30 days sub-acute toxicity study.

| **Hematological parameters (Units)** | **Male** | | | **Female** | | |
| --- | --- | --- | --- | --- | --- | --- |
|  | **Control** | **18KHT01 (mg/kg)** | | **Control** | **18KHT01 (mg/kg)** | |
|  |  | **100** | **500** |  | **100** | **500** |
| WBC (x 10^9^/L) | 3.3 ± 0.9 | 2.48 ± 0.4 | 3.14 ± 0.4 | 3.26 ± 1.0 | 3.58 ± 0.6 | 2.94 ± 0.7 |
| LYM (x 10^9^/L) | 2.52 ± 0.7 | 1.86 ± 0.4 | 2.08 ± 0.4 | 2.58 ± 0.7 | 2.78 ± 0.4 | 2.08 ± 0.5 |
| MID (x 10^9^/L) | 0.12 ± 0.1 | 0.12 ± 0.1 | 0.16 ± 0.1 | 0.12 ± 0.1 | 0.14 ± 0.1 | 0.14 ± 0.1 |
| NEUT (x 10^9^/L) | 0.66 ± 0.2 | 0.5 ± 0.1 | 0.9 ± 0.1* | 0.56 ± 0.3 | 0.66 ± 0.2 | 0.72 ± 0.2 |
| RBC (x 10^12^/L) | 7.09 ± 0.2 | 7.07 ± 0.3 | 7.31 ± 0.3 | 7.25 ± 0.1 | 7.13 ± 0.2 | 7.28 ± 0.5 |
| HGB (g/dL) | 13.08 ± 0.5 | 13.14 ± 0.7 | 13.7 ± 0.8 | 13.56 ± 0.2 | 13.78 ± 0.2 | 13.68 ± 0.6 |
| HCT (%) | 35.08 ± 5.6 | 37.18 ± 2.3 | 38.26 ± 1.6 | 37.78 ± 1.1 | 37.48 ± 2.3 | 37.76 ± 3.0 |
| MCV (fL) | 52.32 ± 1.2 | 52.6 ± 0.9 | 52.36 ± 1.1 | 52.24 ± 1.0 | 52.64 ± 1.9 | 52.02 ± 1.2 |
| MCH (pg) | 18.38 ± 0.4 | 18.52 ± 0.3 | 18.68 ± 0.6 | 18.68 ± 0.3 | 19.3 ± 0.4 | 18.8 ± 0.7 |
| MCHC (g/dL) | 35.24 ± 0.6 | 35.32 ± 0.5 | 35.76 ± 1.8 | 35.86 ± 1.1 | 36.84 ± 2.1 | 36.28 ± 1.6 |
| RDW-SD (fL) | 22.28 ± 1.3 | 21.92 ± 0.9 | 22.28 ± 1.3 | 21.92 ± 0.9 | 22.26 ± 1.9 | 22.66 ± 0.8 |
| RDW-CV (%) | 12.46 ± 0.5 | 12.2 ± 0.4 | 12.46 ± 0.5 | 12.3 ± 0.5 | 12.38 ± 0.8 | 12.76 ± 0.3 |
| PLT (x 10^9^/L) | 288.2 ± 36.6 | 242.2 ± 21.6 | 294.4 ± 48.0 | 252.8 ± 29.5 | 298.6 ± 77.1 | 232.4 ± 38.6 |
| MPV (fL) | 7.24 ± 0.3 | 7.06 ± 0.4 | 7.02 ± 0.2 | 7.16 ± 0.1 | 7.18 ± 0.1 | 6.98 ± 0.1 |
| PDW (%) | 8.9 ± 1.4 | 8.28 ± 0.7 | 9.2 ± 0.5 | 9.16 ± 0.9 | 8.34 ± 0.4 | 9.02 ± 0.7 |
| PCT (%) | 0.20 ± 0.0 | 0.17 ± 0.0 | 0.2 ± 0.0 | 0.18 ± 0.1 | 0.21 ± 0.1 | 0.16 ± 0.0 |
| P-LCR (%) | 9.88 ± 4.4 | 9.34 ± 5.6 | 8.24 ± 2.7 | 8.88 ± 1.6 | 9.54 ± 1.5 | 7.94 ± 0.5 |

Statistical significance was calculated using one-way ANOVA followed by Dunnett's multiple comparisons test. Results are presented as the mean ± standard deviation (n=5) with significance **P*<0.05 vs. controls of respective sex group.

TABLE S6: Comparison of the retention time of marker compounds on standards and 18KHT01.

| **Markers** | **Samples** | **Retention time (min)**  **Mean ± SD** | **RSD (%)** |
| --- | --- | --- | --- |
| Caffeine | Standard | 10.67 ± 0.01 | 0.07 |
|  | 18KHT01 | 10.69 ± 0.02 | 0.19 |
| Epicatechin | Standard | 19.15 ± 0.01 | 0.06 |
|  | 18KHT01 | 19.19 ± 0.03 | 0.13 |
| Corilagin | Standard | 19.99 ± 0.01 | 0.05 |
|  | 18KHT01 | 19.92 ± 0.02 | 0.10 |
| EGCG | Standard | 24.93 ± 0.01 | 0.04 |
|  | 18KHT01 | 24.93 ± 0.02 | 0.08 |
| ECG | Standard | 31.00 ± 0.01 | 0.03 |
|  | 18KHT01 | 31.09 ± 0.02 | 0.07 |
| Ellagic acid | Standard | 33.29 ± 0.02 | 0.05 |
|  | 18KHT01 | 33.28 ± 0.02 | 0.07 |

RSD: Relative standard deviation. Data are presented as mean ±SD (n=4 for standard, and n=6 for 18KHT01).

**Supplemental Figures**


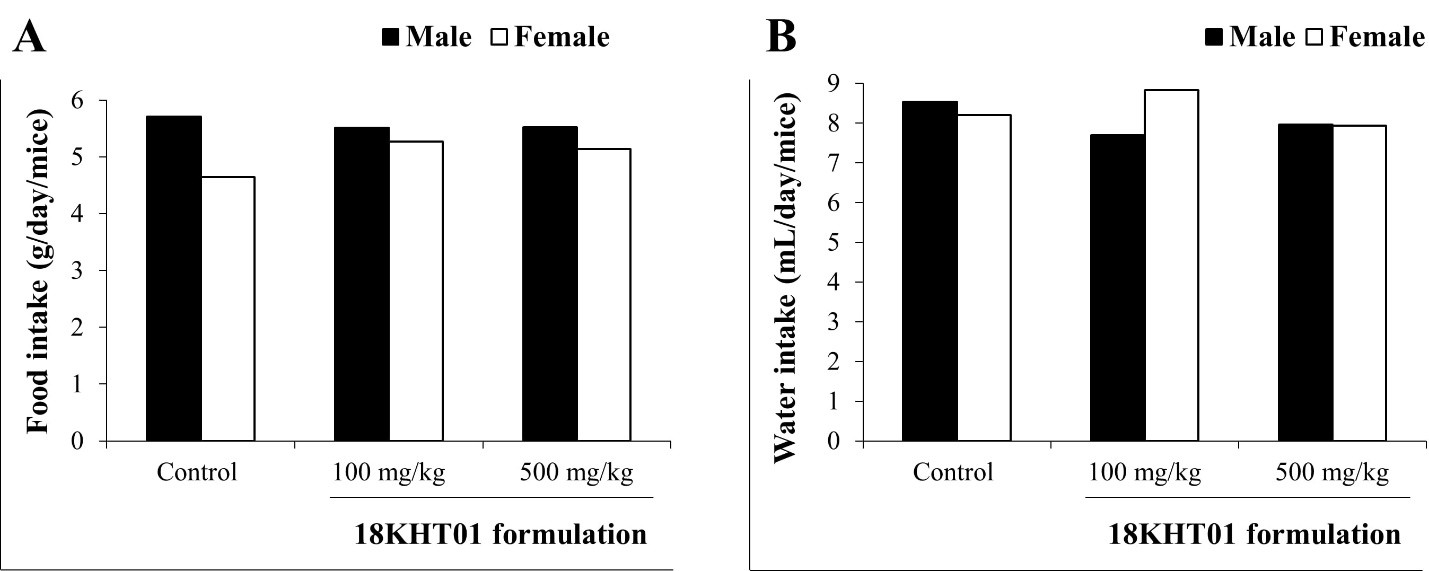


FIGURE S1: Food (A) and water (B) intake pattern in control and 18KHT01 treated male and female mice in 30 days sub-acute toxicity studies.


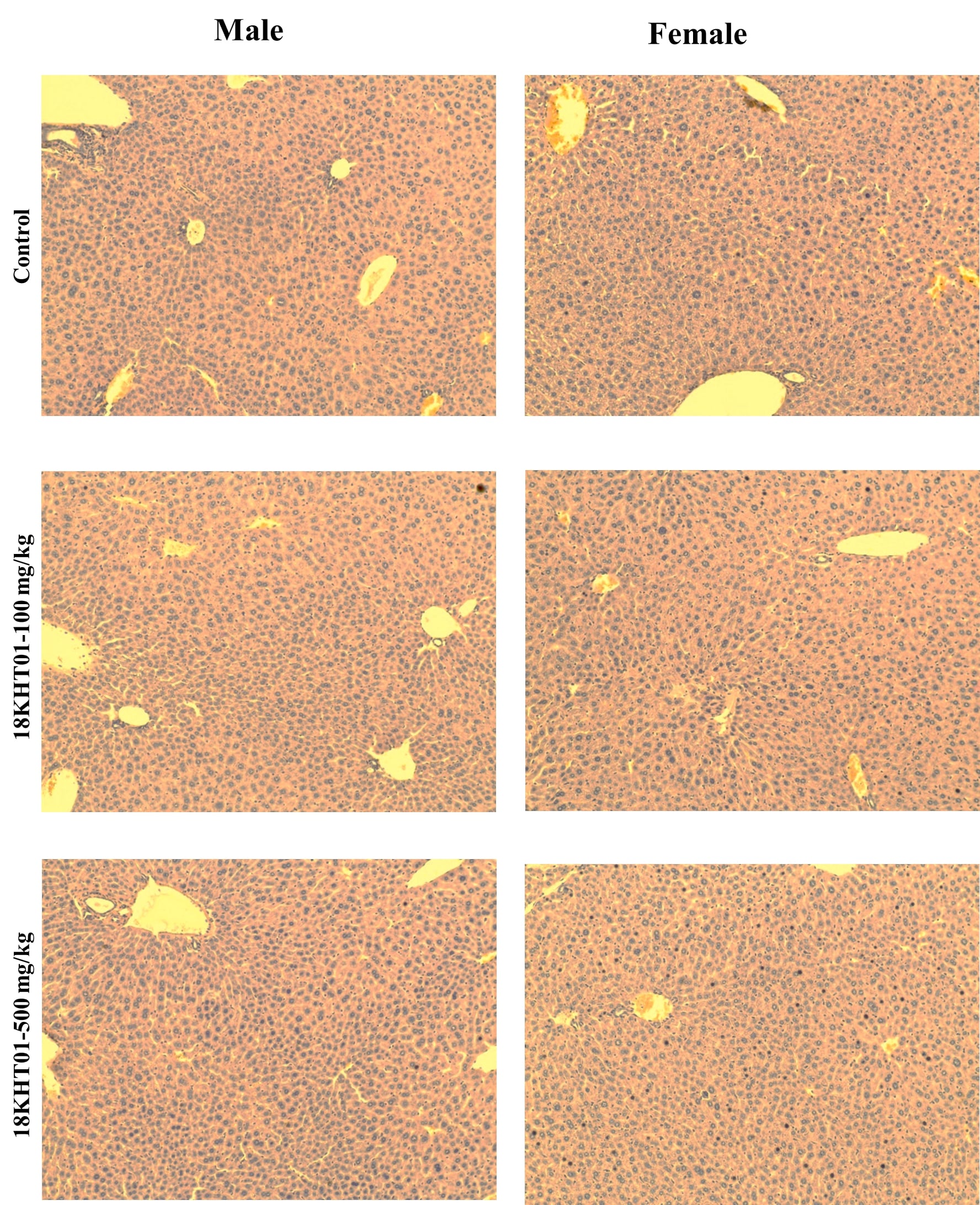


FIGURE S2: Histopathological examination (H&E stain, 20× magnifications) of liver tissue from control and 18KHT01 treated male and female mice. The normal structures of hepatocytes with granulated cytoplasm in control and treated male and female mice are observed.


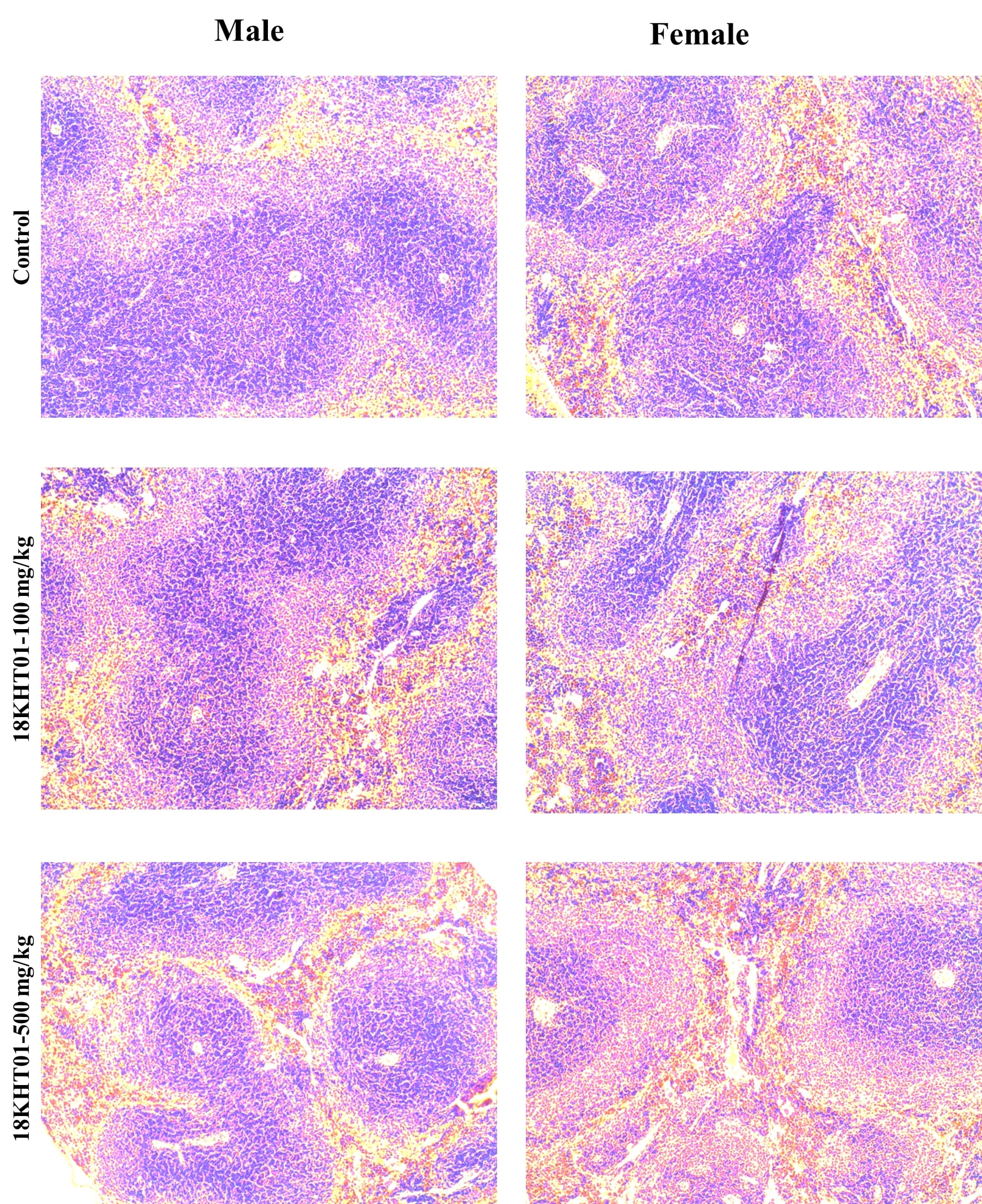


FIGURE S3: Histopathological examination (H&E stain, 20× magnifications) of spleen tissue from control and 18KHT01 treated male and female mice. The normal architectures of white pulp and red pulp in spleen tissues are observed in control and treated groups of either sex.


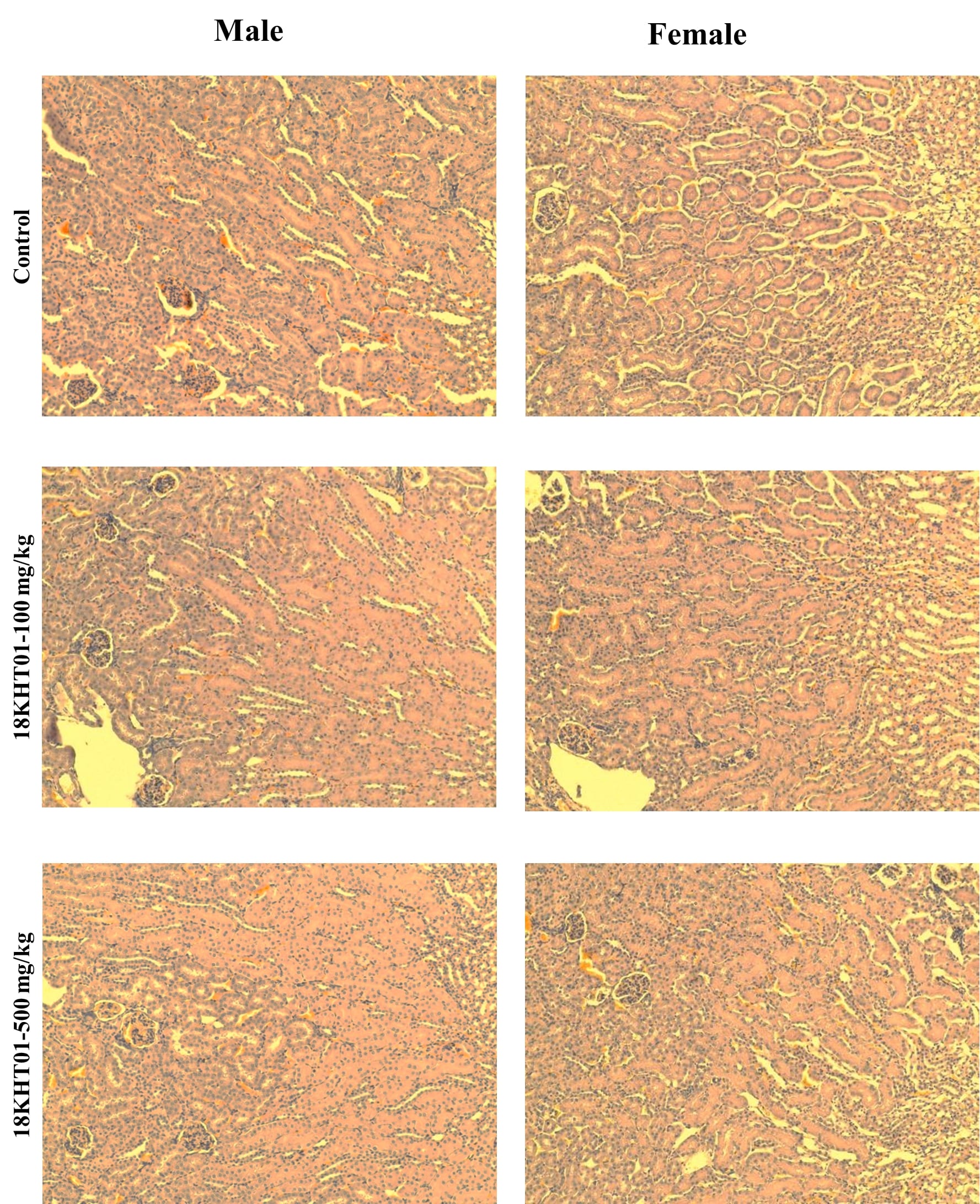


FIGURE S4: Histopathological examination (H&E stain, 20× magnifications) of kidney tissue from control and 18KHT01 treated male and female mice. Normocellular glomeruli with normal tubules and no signs of sclerosis, necrosis or inflammation in 18KHT01 treated male or female mice are observed.


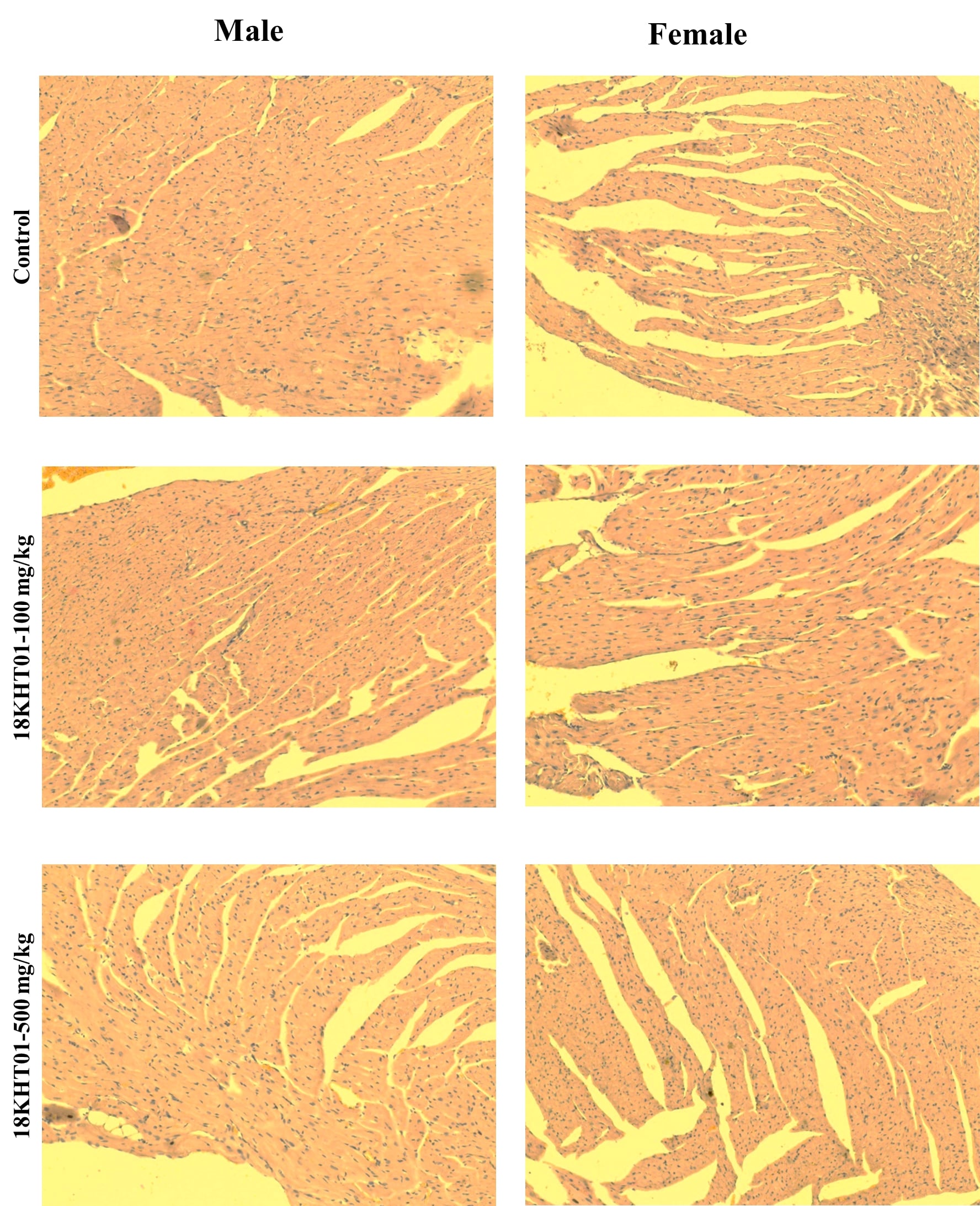


FIGURE S5: Histopathological examination (H&E stain, 20× magnifications) of heart tissue from control and 18KHT01 treated male and female mice. Normal histological observations are seen in control and treated heart specimen.


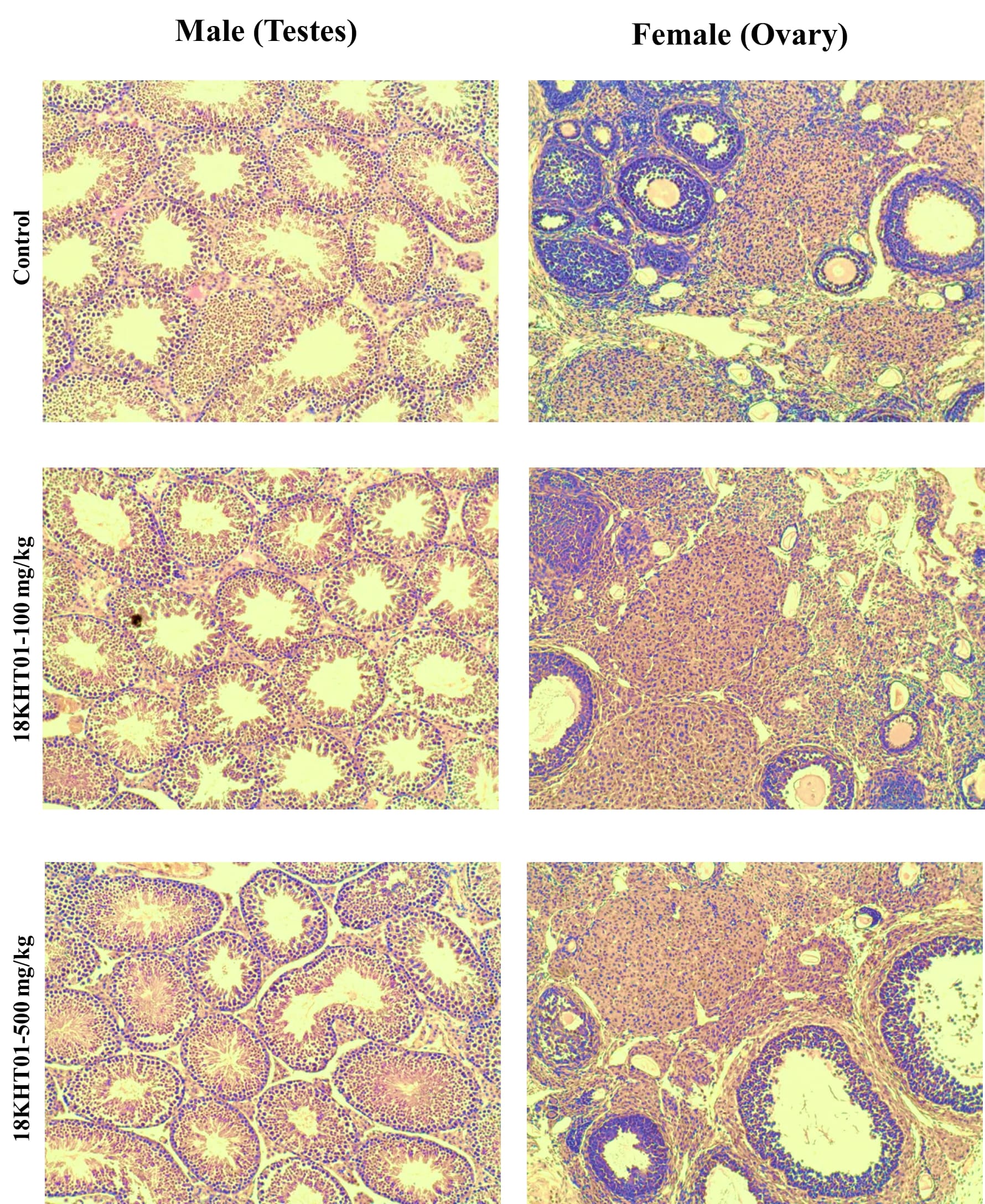


FIGURE S6: Histopathological examination (H&E stain, 20× magnifications) of testes of male and ovary of female control and 18KHT01 treated mice. Testes of male and ovary of female have same symmetry with the normal histo-artitectures in control as well as formulation treated groups.
